# Women with polycystic ovary syndrome (PCOS) have reduced melatonin concentrations in their follicles and have mild sleep disturbances

**DOI:** 10.1186/s12905-022-01661-w

**Published:** 2022-03-21

**Authors:** Hongwanyu Li, Mei Liu, Cong Zhang

**Affiliations:** 1 Center for Reproductive Medicine, Ren Ji Hospital, School of Medicine, Shanghai Jiao Tong University, Shanghai, 200135 China; 2 Shandong Provincial Key Laboratory of Animal Resistance Biology, College of Life Sciences, Shandong Normal University, Jinan, Shandong China; 3 Shanghai Key Laboratory for Assisted Reproduction and Reproductive Genetics, Shanghai, China; 4 Department of Obstetrics, Affiliated Hospital of Shandong University of Traditional Chinese Medicine, No. 42 Wenhua xi Road, Jinan, 250011 Shandong China

**Keywords:** Melatonin, Polycystic ovarian syndrome, Follicular fluid, Circadian rhythm; Sleep quality

## Abstract

**Background:**

Polycystic ovary syndrome (PCOS) is a common gynecologic disorder related to abnormal circadian rhythm. Therefore, we aimed to find whether the level of melatonin, a rhythm regulating hormone changed in the ovarian microenvironment in this disease.

**Methods:**

The melatonin concentrations in follicular fluid (FF) were measured in 35 PCOS and 36 non-PCOS women undergoing in vitro fertilization (IVF) treatment.

**Results:**

The FF melatonin concentration was significantly lower in PCOS women than non-PCOS women (*p* = 0.045) and it was found positively correlated with serum basal FSH level (r = 0.308, *p* = 0.013). In IVF procedures, there was no significant difference in the fertilization rate of oocytes between the two groups, but the high-quality embryogenesis rate on the third day of the PCOS group was significantly lower than that of the control group (*p* = 0.042), which showed a weak positive correlation with the FF melatonin concentration (r_s_ = 0.240, *p* = 0.044). Furthermore, there was no significant difference in overall pregnancy outcome. The PSQI questionnaire showed that sleep disorders were more likely to exist in the PCOS group, though there was no significant difference.

**Conclusion:**

The obtained results suggested PCOS women had lower melatonin concentrations in the ovarian microenvironment.

## Introduction

Circadian rhythm is an oscillation phenomenon with a cycle of about 24 h, also known as biological clock. This clock transforms the light–dark cycle of the environment to endogenous rhythmic signals in vivo and optimize the time sequence of life activities. In mammals, the suprachiasmatic nucleus (SCN) of the hypothalamus is the regulatory center of the circadian rhythm. SCN receives light signals from the retina, and synchronizes the rhythms of surrounding tissues and organs through body fluid and neuromodulation [1]. The function of female reproduction depends on the synchronization of the biological clock signal of the hypothalamus-pituitary-ovarian (HPO) axis. When the central SCN is not synchronized with the ovarian circadian clock, normal ovarian function would be affected. Demographic data show that staying up for a long time, shift work and flying across time zones can cause women's circadian rhythm disorders, which are related to menstrual disorders and adverse pregnancy outcomes [2].

Melatonin (N-acetyl-5-methoxytrypamine), an indoleamine hormone mainly produced by pineal gland, is an important mediator of SCN to regulate the rhythm of peripheral organs [3]. The activity of SCN is inhibited at night, and the norepinephrine produced by the upper cervical ganglia increases, which stimulates the synthesis of melatonin in the pineal gland, thereby promoting sleep in other nocturnal rhythms. In contrast, the synthesis of melatonin is inhibited on the day that light activates SCN. In addition, the surrounding reproductive cells, including granulosa cells, cumulus and oocytes also have the ability to secrete melatonin [4].

Melatonin regulates the reproductive system in a central and peripheral manner. As a systemic hormone modulator, melatonin can affect sexual maturity and reproductive function by activating its receptor and the binding site of the HPO axis [5–7]. On the other hand, the direct effect of melatonin on the ovaries has also received widespread attention. Melatonin in follicle is from serum and the surrounding reproductive cells, therefore, the concentration in the follicular fluid becomes much higher than that in the blood [8]. As follicles develop, the melatonin concentration in the follicular fluid gradually increases and reaches a peak before ovulation [9]. It has been proved that melatonin can function as a free radical scavenger to reduce oxidative stress in local tissue [10, 11], so it has the potential to support follicular development by stabilizing the local ovarian microenvironment.

Polycystic ovary syndrome (PCOS) is considered to be one of the most common reproductive endocrine diseases, and about 10% of women suffer from the disease during childbearing age [12]. The most typical clinical manifestation of PCOS are ovulation dysfunction (oligomenorrhea or amenorrhea), hyperandrogenism, and polycystic ovarian morphology. Besides metabolic disorder like insulin resistance is also common problems in PCOS patients. It is worth noting that PCOS patients are more likely to suffer from sleep disorders such as drowsiness and obstructive sleep apnea [13]. Sleep monitoring also showed that the sleep structure of PCOS patients was different from that of their normal peers [14]. Recently, many evidences have further confirmed the relationship between circadian rhythm disorders and PCOS, as well as related sleep, metabolic and reproductive dysfunctions [15]. A cross-sectional study showed that the circadian rhythm of PCOS patients was characterized by delayed offset of melatonin relative to clock time and sleep time, and this delay was further found to be associated with the higher serum free testosterone levels and worse insulin sensitivity [16]. Ovarian dysfunction is another serious problem in PCOS patients. The growth of follicles in women with PCOS is arrested at the early antral stage [17]. In this case, PCOS is one of the main causes of anovulatory infertility after menarche. Women with PCOS take longer to achieve their first delivery [18]. Assisted reproductive technology (ART) is usually the final means of obtaining pregnancy for PCOS patients. Most current studies have shown that the serum melatonin concentration of PCOS women is higher than that of healthy women [19, 20], supported by the higher concentration of the direct metabolite of melatonin α-MT6s in the urine of PCOS women [21, 22]. At the same time, the circadian difference in serum melatonin concentration in PCOS patients is smaller than that in normal people, maybe due to disrupted circadian rhythm [19]. Like in serum, circadian and circannual variations in the concentration of melatonin in follicular fluid were also found [23], which indicates that the effect of arrhythmia on PCOS patients may also exist at the follicular level. However, there are very few reports on the level of melatonin in follicular fluid, which limits our understanding of the role of melatonin in PCOS. At the same time, the follicular fluid provides an external environment for the development and maturation of the oocytes. The melatonin in the follicular fluid may be directly related to the maturation and ovulation disorders of the oocytes of PCOS patients. Therefore, we want to know whether there is a correlation between the rhythm disorder represented by sleep arousal disorder, ovarian dysfunction and the change of melatonin level in the ovarian microenvironment, so that we can evaluate the therapeutic effect of melatonin on PCOS infertility. In this study we collected a cohorts of women receiving IVF treatment for PCOS and non-PCOS reasons, compared the difference in melatonin concentration in their follicles, and determined whether the melatonin level in the follicles was related to the quality of sleep and embryo development by evaluating IVF and pregnancy results. This study provides a new perspective for us to understand the relationship between rhythm disorders and ovarian function in PCOS women.

## Materials and methods

### Subjects

A total of 71 women aged 25 to 35 were enrolled in this study, including 35 PCOS women and 36 controls. They all underwent in vitro fertilization (IVF) or intracytoplasmic sperm injection (ICSI) treatment at Renji Hospital's Reproductive Medicine Center from October 2018 to December 2018. The study was approved by the Reproductive Ethics Committee of Renji Hospital, and informed consents were obtained from all subjects. PCOS was diagnosed according to the Rotterdam PCOS Consensus Standard (2003). Women in the control group were infertile due to tubes or male factors with normal levels of sex hormones and anti-Mullerian hormone (AMH). All women had not taken oral contraceptives for at least three months. Exclusion criteria include congenital uterine abnormalities, gynecological tumors and other endocrine diseases, such as hyperthyroidism, hypothyroidism, hyperprolactinemia, Cushing’s syndrome, etc. Basic information about the participants was collected at the first visit, including age, the duration of infertility and the cause of infertility.

### Serum sample collection and hormone profiles determination

Peripheral blood serum samples were collected on the second to third days of the menstrual cycle. Use Roche Cobas e601 automatic electrochemiluminescence immunoassay analyzer (ECLIA) to determine serum basic hormone levels, including follicle stimulating hormone (FSH), luteinizing hormone (LH), estradiol (E2), progesterone (P), and testosterone (T), and determine the AMH level with an enzyme-linked immunosorbent assay kit (Kangrun Biotechnology Co., Ltd., Guangzhou).

### Follicular fluid samples and melatonin determination

Follicular fluid samples were obtained on the day of retrieving oocyte day during 8:30am to 10:00 am by follicle puncture under transvaginal ultrasound guidance. Follicular fluid of both groups was collected from the dominant follicle with a diameter greater than 18 mm. The follicular fluid in both groups was obtained from the dominant follicle with a diameter greater than 18 mm.After collection, the follicular fluid was centrifuged at 1000 g for 10 min to get rid of a small amount of cell components and all the samples were stored at − 80 °C until determination. All the steps were carried out under the dark condition. Melatonin concentration was measured by ^125^I-radioimmunoassay with a RIA kit from Beijing sinouk institute of biological technology. The determination range was 0–400 pg/ml. The intra and inter assay CV were < 4.5% and < 8.5% respectively. The average recovery rate was 97.5%.

### In vitro fertilization therapy

Once entering the IVF procedures, each patient was provided a stimulating protocol of controlled ovarian hyperstimulation. The protocol was chosen from long agonist protocol, short agonist protocol, antagonist protocol or minimal stimulation protocol by the attending doctor according to the assessment of ovarian function and the difference of these protocols was mainly using of exogenous gonadotropin (GN) drugs including FSH and human menopausal gonadotropin. Every two to three days after the initiation of ovulation induction, the patient underwent an ultrasound examination to assess follicular growth, and serum E2, FSH, LH and progesterone was tested at a later stage. Until the leading follicle reached 20 mm and the serum E2 level match with the number of mature follicles, patients received hCG or triptorelin acetate injection the choice and dosage of which was determined according to the E2 level and the number of mature follicles. Ultrasound guided puncture was performed in the next 33–36 h followed by the IVF. For some couples with severe male factors, ICSI was considered for requirement. Three days after artificial insemination, the cleavage embryos were evaluated, high-quality embryos were selected and at most four of them were used for fresh embryo transfer or cryopreservation as the rest continued to develop into blastocysts on the fifth or sixth day before the qualified ones got cryopreserved. The cleavage embryos and blastocysts was evaluated by the system of Balaban [24] and the system of Gardener and Schlenker [25]. The patients with embryos obtained then entered the transfer cycle, and at most two fresh embryos or frozen embryos could be transferred at one time. After transplantation, patients received serum β—hCG and ultrasound examination to determine if they got pregnant. Pregnant women were followed up by the Center until delivery to know their pregnancy outcomes.

### Sleep quality analysis

Pittsburgh Sleep Quality Index (PSQI) questionnaire was completed by 17 PCOS women and 24 non-PCOS women after their oocyte retrieval to estimate the sleep quality. The scale was scored by seven components, including subjective sleep quality, sleep latency, sleep duration, habitual sleep efficiency, sleep disorder, use of sleep drugs, daytime dysfunction. Each component was graded from 0 to 3 according to the severity of the disorder, with a total of 21 points. Women with 5 or more points were regarded as poor sleep quality.

### Statistical analysis

The statistical analysis was completed by using SPSS (version 25, IBM Corporation, USA). The variables were evaluated by descriptive statistical methods and normality tests (Kolmogorov–Smirnov test). The skewed variables were normalized by logarithmic transformation or square root operation. Normally distributed variables were expressed as mean ± SEM. Skew variables that cannot be normalized were expressed as the medians (interquartile range). In order to compare the parameters between the two groups, the independent sample T test was used for normally distributed continuous variables, and the Mann–Whitney U test was used for skewed continuous variables. Pearson correlation test was used to assess the correlation between continuous normal variables, otherwise Spearman correlation test was exploited. The difference between two constituent ratios was tested by χ^2^ or Fisher's exact test. A two-sided *p* value of < 0.05 was considered statistically significant.

## Results

### Clinical characteristics and hormonal profiles

**Fig. 1:**
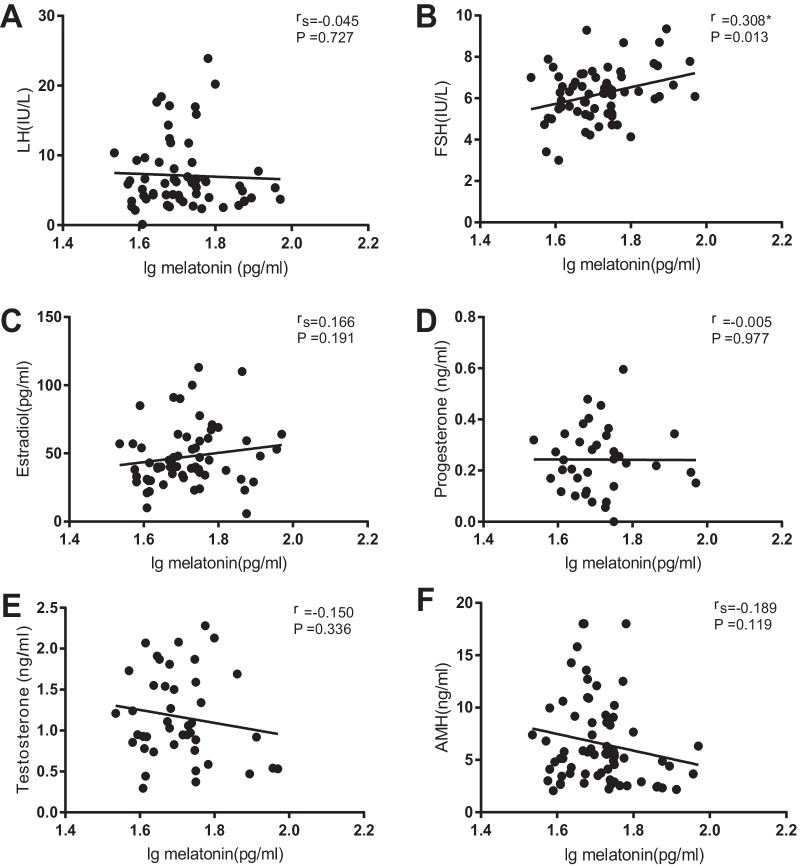
Correlation analysis between melatonin in follicular fluid and basic hormones in serum. A significant positive correlation between the melatonin in follicular fluid and serum FSH (r = 0.308; *p* = 0.013). No significant correlation was found between the melatonin concentration and LH (rs = − 0.045; *p* = 0.727), Estradiol (rs = 0.166; *p* = 0.191), progesterone (r = − 0.005; *p* = 0.977), testosterone (r = − 0.150; *p* = 0.336) and anti Mullerian hormone (rs = − 0.189; *p* = 0.119). *r represents Pearson correlation coefficients; rs represents Spearman rank correlation coefficients

The clinical characteristics and hormone profiles of the control group and PCOS group are given (Table 1). There were no significant differences in age, years of infertility and BMI between the two groups. As for serum hormone levels, the estradiol, LH and LH/FSH ratios of the PCOS group were significantly higher than those of the control group. The testosterone and AMH concentrations in the PCOS group were also higher. At the same time, it was found that there was no significant difference in the concentration of FSH and progesterone. On the contrary, the concentration of melatonin in the follicular fluid of the PCOS group was significantly lower than that of the control group (Table 1). Furthermore, the correlation between melatonin concentration and hormones was also analyzed (Fig. 1). We found that the melatonin concentration in follicular fluid was significantly positively correlated with the basal FSH level (r = 0.308, *p* = 0.013). However, there was no correlation between other hormones and melatonin concentration.

**Table 1:** Clinical characteristics and hormonal profiles of the control and polycystic ovarian syndrome (PCOS) group

	Controls **(**n = 36**)**	PCOS **(**n = 35**)**	*p* value
Age (years)	28.063 ± 1.000	26.926 ± 1.000	0.094
Infertility years (years)	3 (2–4)	2 (1–4)	0.280
BMI (kg/m^2^)	21.134 ± 0.081	22.333 ± 0.154	0.119
FSH (IU/L)	6.216 ± 1.602	5.998 ± 1.193	0.542
LH (IU/L)	4.085 (3.235–5.773)	8.090 (5.940–15.110)	< 0.001***
LH/FSH	0.636 ± 1.907	1.462 ± 1.757	< 0.001***
Estradiol (pg/ml)	38.845 (30.750–55.250)	47.000 (39.598–69.695)	0.048*
Progesterone (ng/ml)	0.239 ± 0.140	0.248 ± 0.029	0.837
Testosterone (ng/ml)	0.914 ± 0.441	1.407 ± 0.519	0.020*
AMH (ng/ml)	3.506 (2.650–4.813)	8.825 (5.868–12.190)	< 0.001***
Intrafollicular Melatonin (pg/ml)	54.455 ± 1.303	49.137 ± 1.151	0.045*

*p < 0.05; ***p < 0.001

### Comparison of IVF outcomes

The IVF outcomes of the two groups were then compared (Table 2). The total number of oocytes, MII oocytes, fertilized oocytes, high-quality embryos and high-quality blastocysts in the PCOS group were relatively large. Then, we further compared the rates. No significant difference was found in the ratio of MII oocytes. The rate of PCOS fertilized eggs was found to be higher in the IVF procedure, but due to the male sterility of the control group, there was no difference in the ICSI procedure. However, the incidence of quality embryos on D3 in the PCOS group was lower than that in the control group. It was also found that there was a weak positive correlation between the melatonin concentration and the ratio of high-quality embryos on D3 (rs = 0.240, *p* = 0.044). After selection on D3, there was no significant difference between the blastocyst formation rate of D5 or D6 and the percentage of high-quality blastocysts (Table 2).

**Table 2:** IVF outcomes of the control and polycystic ovarian syndrome (PCOS) group

	Controls **(**n = 36**)**	PCOS **(**n = 35**)**	P value
No. of oocytes retrieved	13.00 **(**9.25–16.00**)**	19.00 **(**15.00–24.00**)**	< 0.001^***^
Rate of MII oocytes	85.6% (74.8–92.5%)	92.8% (72.0–100.0%)	0.143
IVF fertilization rate	53.7% ± 3.92% (n = 27)	65.7% ± 4.1% (n = 30)	0.011^*^
ICSI fertilization rate	71.1% ± 5.94% (n = 12)	59.1% ± 7.4% (n = 5)	0.255
Rate of top quality embryos on D3	87.0% (57.1–100.0%)	73.1% (47.1–87.5%)	0.042^*^
Rate of top quality blastocysts on D5/6	75.0% (50.0–100.0%)	64.6% (46.0–82.5%)	0.185

IVF, in vitro fertilization; ICSI, Intracytoplasmic sperm injection; *p < 0.05; ***p < 0.001

Rate of MII oocytes was based on the number of MII oocytes/the number of oocytes retrieved × 100%

Rate of oocytes fertilized was based on the number of oocytes fertilized/ the number of oocytes retrieved × 100%

ICSI was performed in 3 cycles and the rate was separately calculated

Rate of top quality embryos on D3 was based on the number of top quality embryos on D3/ the number of oocytes normal fertilized (oocytes with two pronucleus) × 100%

Rate of top quality blastocysts on D5/D6 was based on the rate of top quality blastocysts/the number of blastocysts continued to be cultured on D3 × 100%

### Comparison of pregnancy outcomes

Similar results were found in the follow-up after translation. Among the enrolled participants, 33 PCOS women and 35 women entered the transplantation cycle, and some women experienced multiple cycle of transplants. In 48 control cycles and 40 PCOS cycles, we found no significant difference in embryo implantation success rate, clinical pregnancy rate, early miscarriage rate (less than 12 weeks) and live birth rate between the two groups (Table 3). There was no significant difference in melatonin concentration between pregnant women and non-pregnant women.

**Table 3:** Transplantation and pregnancy outcomes of the control and polycystic ovarian syndrome (PCOS) group

	Control (n = 48)	PCOS (n = 40)	P value
Rate of clinical pregnancy	62.5% (30/48)	77.5% (31/40)	0.129
Rate of implantation	59.3% (35/59)	66.7% (32/48)	0.435
Rate of early abortion	6.7% (2/28)	0.0% (0/31)	0.238^a^
Rate of live birth	58.3% (28/48)	77.5% (31/40)	0.057

^a^Fisher exact test is used

Rate of clinical pregnancy was based on the number of cycles with ultrasound diagnosis of clinical pregnancy/ the number of transfer cycles

Rate of implantation was based on the number of embryo sacs under ultrasound diagnosis /the number of embryo transferred (one or two embryos was transferred per cycle)

Rate of early abortion was based on the number of cycles with abortion in 12 weeks/ the number of cycles with clinical pregnancy

Rate of live birth was based on the number of cycles with live birth/ the number of transfer cycles

### Sleep quality analysis

PCOS women tended to have a higher rate of sleep disorders as more of them had high PSQI scores, though not significant (Table 4). Specifically, the there was no significant difference in the rate of self-reported sleep disorders. At the same time, the ratio of daytime sleepiness due to sleep deprivation was also similar between the two groups. However, the PCOS women had significantly less sleep time during night (*p* = 0.027). What's more, their lower habitual sleep efficiency (*p* = 0.012) made the situation even worse. There were also more women having sleep structure disturbance in PCOS group (*p* = 0.049). But when the relationship between the melatonin concentration and evaluation index in the questionnaire analyzed, no significant correlation was found.

**Table 4:** Sleep quality of the control and polycystic ovarian syndrome (PCOS) group

	Control (n = 24)	PCOS (n = 17)	*p* value
Subjective bad sleep quality	58.3% (14/24)	76.5% (13/17)	0.227
Sleep duration(h)	8.46 ± 1.00	7.74 ± 0.93	0.027*
Habitual sleep efficiency	94.5% (88.3–98.5%)	86.3% (81.2–91.7%)	0.012*
Sleep structure disturbance	45.8% (11/24)	76.5% (13/17)	0.049*
Daytime dysfunction	54.2% (13/24)	64.7% (11/17)	0.500
Total PSQI ≥ 5	25.0% (6/24)	41.2% (7/17)	0.273

PSQI: Pittsburgh sleep quality index;*p < 0.05

## Discussion

In this study, we measured the concentration of melatonin in the follicular fluid of the leading follicles of PCOS and controls receiving IVF treatment, and collected their sleep quality information through the PSQI questionnaire. In addition, we analyzed the correlation between melatonin and serum hormones, embryonic development, reproductive outcomes and sleep quality. Our research showed that the concentration of melatonin in the follicular fluid of PCOS women and the efficiency of habitual sleep were lower than those of controls, and the sleep structure of PCOS women was disordered. The incidence of top embryos on the third day of PCOS women was lower than that of the control group, and there was a weak positive correlation between the incidence and concentration of melatonin. In addition, we found that the concentration of melatonin in follicular fluid was positively correlated with serum basal FSH levels (r = 0.308, *p* < 0.05).

In recent years, dysrhythmia is believed to affect the ovarian function and is one of the pathogenesis of PCOS [26, 27]. Melatonin is likely to be involved in this process. Although there is still controversy, most of the available evidence supports that the serum melatonin concentration of PCOS women is higher than that of the control group [19, 20]. Generally, the concentration of melatonin in follicular fluid is positively correlated with serum [8]. However, in PCOS women, this correlation seems to be challenged. Previous studies have found that the concentration of melatonin in the follicular fluid in the small follicles (< 10 cm) of PCOS women is significantly lower than that in the large follicles (> 18 cm) of non-PCOS women [28], but there is significant difference between the melatonin levels in these two size of follicles [9], which may affect the assay. Therefore, in our study, we chose the large anterior follicles that played a more important role in the clinical oocyte collection of the two groups. It showed that the concentration of melatonin in the follicular fluid of PCOS women was still lower than that of the normal control group, although not as significant as previous studies [9, 28]. The decrease of melatonin concentration in follicular fluid may come from two aspects: one was the decrease of absorption from the serum; the other was the decrease in ovarian secretion caused by follicular atresia in PCOS women.

In our cohort, we found that women with PCOS did show more severe sleep disorders than the control group, especially in terms of reduced absolute sleep time and habitual sleep efficiency. They were also more likely to suffer self-reported sleep disorders, which is consistent with previous report [29], although this result was not significant, it may be because both groups were in the preoperative preparation for the upcoming test-tube cycle for egg retrieval surgery which might make them feel anxious and consequently affected their sleep. In addition, the night sleep of PCOS women was more likely to be affected, which was manifested as a disorder of sleep structure. It made them more likely to be exposed to light at night, thereby inhibiting the production of melatonin. However, we failed to find a direct relationship between melatonin concentrations in follicular fluid and sleep scale indicators, which is very similar to the analysis of serum melatonin concentration [30]. One possible explanation is that the relationship between melatonin and sleep is precise and complex. Dynamic melatonin monitoring can help us better understand this problem, but because of the difficulty in obtaining follicular fluid, our research could not do this.

As we all know, melatonin acts as an antioxidant and free radical scavenger. Oxidation-antioxidant balance plays an important role in oocyte maturation. Physiological level of reactive oxygen species (ROS) can be used as the second messenger of genes related to oocyte maturation to regulate the function of oocytes, but excessive accumulation of ROS can cause oxidative stress, which will adversely affect ovarian function [31]. In the ovary, this antioxidant plays a variety of roles, such as inhibiting cell apoptosis, reducing autophagy to improve mitochondrial function, and maintaining telomeres to delay ovarian senescence [32–34]. Therefore, the lack of melatonin would lead to a decrease in antioxidant capacity in the follicular microenvironment. The results showed that the levels of ROS in follicular fluid and granulosa cells of PCOS women were higher than those in the control group, leading to follicular development disorders in PCOS women [35, 36]. Our research showed that despite the similar fertilization rate, PCOS women with lower levels of melatonin in the follicular fluid had more difficulty obtaining high-quality embryos at the 8-cell stage. After effective selection, no significant differences were found in embryonic development, transplantation and subsequent stages of pregnancy, indicating that the effect of melatonin on oocytes may affect early embryonic development. As a result, the overall results of IVF between the two groups were similar, which is consistent with previous knowledge [17].

Interestingly, in our results, we also found that the concentration of melatonin in the follicular fluid was positively correlated with serum basic FSH. During the fetal period, primordial follicles are formed. FSH receptor are expressed in follicles from primary stages, promote the growth of preantral follicles and antral follicles, and also protect antral follicles from atresia [37]. Elevated levels of FSH in preantral or early antral follicles inhibit AMH expression, favoring CYP19A1 aromatase expression and E2 production [38]. The follicular development of PCOS patients is arrested. We infer that melatonin might be involved in the regulation of HPO axis by affecting the expression of FSH, and thus the regulation of follicular growth.

The biological clock plays an important role in life activities. Light is the strongest regulator of biological rhythms. From the fetal period, the secretion of melatonin is adjusted with the changes in the light and dark cycle, thereby transmitting the biological clock information. Compared with the natural clock, delayed light at night will delay the secretion of melatonin, which can lead to a series of health problems [39]. Unfortunately, modern people seem to be unable to avoid the lights at night. In industrial society, 20% of people work abnormally, such as shift or night shift, which puts them in an abnormal light–dark cycle [40]. More seriously, even low-intensity light can weaken the secretion of melatonin. In other words, the very common light sources in the living environment, namely LED lights, mobile phones, computers and other equipment will also cause this disease [41]. These factors together lead to the so-called social time difference, that is, the imbalance between natural light time and social time. The subsequent delay in the secretion of melatonin may lead to a feedback increase in the melatonin concentration in the ovarian environment, and ultimately, through the regulation of sex hormones and oxidation-antioxidation balance, the fertility of contemporary women of childbearing age will decline. Women with PCOS have a delay similar to the aforementioned melatonin secretion, and their nighttime sleep structure abnormalities further increase the possibility of night exposure, which forms a vicious circle. This may help explain the increasing incidence of the disease in nowadays. Our results further prove the positive role of melatonin in the clinical treatment of infertility in PCOS women. On the one hand, melatonin tends to make it easier for PCOS women to become pregnant naturally. A previous clinical study found that oral melatonin can significantly reduce serum androgen levels and AMH, increase FSH and improve the menstrual cycle [42]. Moreover, melatonin may promote oocyte and embryo maturation during the necessary IVF treatment. Studies have shown that the addition of melatonin in vitro culture medium can improve oocyte maturity and pregnancy rate in PCOS women [28, 43]. Although the safety of exogenous melatonin has been widely recognized, and long-term continuous melatonin therapy has been used for many diseases [44–46], previous studies have also pointed out that melatonin therapy can increase the induction of ovarian hyperstimulation (OHSS) risk [47, 48]. Therefore, the application of melatonin in the clinical treatment of assisted reproduction needs further exploration.

The limitation of this study is that due to the fixed operation time, we can only collect follicular fluid in a single time. Therefore, we have not analyzed the circadian rhythm of melatonin in the follicular fluid of the two groups. In addition, since the concentration of melatonin in serum and follicular fluid is different, simultaneous determination of melatonin and sex hormone levels in serum and follicular fluid may provide more information for further regulation of melatonin.

## Conclusion

In this study, we measured the melatonin concentrations in a cohort of PCOS and non-PCOS women to help explore the melatonin-sleep paradigm and follicular development in PCOS. We showed lower melatonin concentrations of large leading follicles and mild sleep disturbances in PCOS women. In view of the complexity of melatonin regulating sleep and ovarian function, large-scale basic research on clinical and pathophysiological mechanisms is needed.

## Data Availability

The datasets used or analysed during the current study are available from the corresponding author on reasonable request.
